# Control of lymphocyte functions by gut microbiota-derived short-chain fatty acids

**DOI:** 10.1038/s41423-020-00625-0

**Published:** 2021-04-13

**Authors:** Chang H. Kim

**Affiliations:** Department of Pathology and Mary H. Weiser Food Allergy Center, University of Michigan School of Medicine, Ann Arbor, MI USA

**Keywords:** Microbiota, Dietary fiber, Short-chain fatty acids, Innate lymphoid cells, B cells, Th1, Th17, Tregs, CD8, Microbial metabolites, Mucosal immunology, T-helper 17 cells

## Abstract

A mounting body of evidence indicates that dietary fiber (DF) metabolites produced by commensal bacteria play essential roles in balancing the immune system. DF, considered nonessential nutrients in the past, is now considered to be necessary to maintain adequate levels of immunity and suppress inflammatory and allergic responses. Short-chain fatty acids (SCFAs), such as acetate, propionate, and butyrate, are the major DF metabolites and mostly produced by specialized commensal bacteria that are capable of breaking down DF into simpler saccharides and further metabolizing the saccharides into SCFAs. SCFAs act on many cell types to regulate a number of important biological processes, including host metabolism, intestinal functions, and immunity system. This review specifically highlights the regulatory functions of DF and SCFAs in the immune system with a focus on major innate and adaptive lymphocytes. Current information regarding how SCFAs regulate innate lymphoid cells, T helper cells, cytotoxic T cells, and B cells and how these functions impact immunity, inflammation, and allergic responses are discussed.

## Introduction

**Fig. 1:**
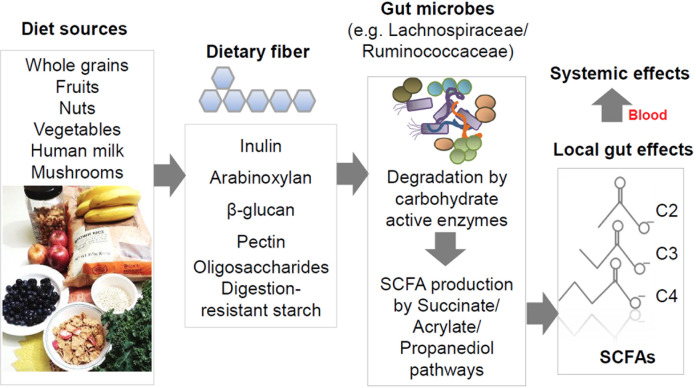
Major prebiotic sources and production of SCFAs. SCFAs, such as acetate (C2), propionate (C3), and butyrate (C4), are produced from a number of DF and digestion-resistant starches by the cooperative catabolic activity of commensal bacteria. These bacteria have complex carbohydrate-degrading enzymes and/or the enzymes involved in SCFA-producing pathways, such as the succinate, acrylate, and propanediol pathways. Food rich in DF enhances the growth of the commensal bacteria that produce SCFAs. Whole grains are a good source of inulin, arabinoxylan, and β-glucan. Fruits are a good source of pectin. Human breast milk is a rich source of oligofructose, which is used to produce SCFAs in infants. Starches engineered to be resistant to digestion also reach the colon for microbial fermentation. Inadequate DF consumption is common in certain demographic groups in developed countries, leading to SCFA deficiency-related immune insufficiency and dysregulation. Produced SCFAs have strong local effects on the intestine and can exert systemic effects following transport to other organs through the portal vein and blood circulatory system

The colon and the adjacent part of the small intestine contain many microbes, which are predominantly bacteria and some fungal species. These microbes produce a myriad of metabolites from dietary components and host-produced biomolecules in the gut.^1,2^ Some of these metabolites function as important regulators of host physiology and the immune system. Short-chain fatty acids (SCFAs), such as acetate (C2), propionate (C3), and butyrate (C4), may be the best examples of these biologically active microbial metabolites. SCFAs are efficiently produced by certain bacterial species in the Firmicutes and Bacteroidetes phyla (Fig. 1). As the most abundant anions in the colonic lumen, SCFAs are absorbed and partially utilized by colonic epithelial cells. They reach other organs and exert regulatory functions to control glucose and fat metabolism; they also regulate the immune system. SCFAs promote immunity and suppress inflammatory responses in the intestine and other organs. These functions are mediated by multiple mechanisms, including histone deacetylase (HDAC) inhibition, G-protein-coupled receptor (GPR) signaling, acetyl-CoA production, and metabolic integration. Through combinations of these mechanisms, SCFAs can promote both immune responses and immune tolerance. In this review, the functions of SCFAs and their receptors in regulating immune cells, with a focus on innate lymphoid cells (ILCs), T cells, and B cells, are discussed.

## Overview of the immunoregulatory effects of SCFAs

In general, the available body of evidence indicates that SCFAs enhance immunity to defend against pathogens. In experimental animals, SCFAs increase immunity to extracellular bacteria (*C. rodentium* and *C. difficile*), viruses (influenza and respiratory syncytial viruses), and intracellular pathogens (*Listeria monocytogenes* and *Salmonella typhimurium*).^3–6^ In contrast, DF and SCFAs exacerbate helminth infection.^7^ Whether this is a universal phenomenon or specific to certain helminth types remains to be established.

DF and SCFAs have protective effects on allergic diseases.^8–11^ DF and SCFAs suppress allergic responses in the lungs in experimental animals and are linked to decreased allergic responses in humans. DF and SCFAs are also implicated in suppression of experimental food allergy in animals.^10^ As discussed later in this article, SCFAs suppress ILC2 and IgE responses,^10,12,13^ and these effects, in part, account for the observed beneficial effects on allergic diseases.

High levels of DF intake and SCFA production decrease colitis in animals and human patients.^14–16^ SCFA administration ameliorates various types of experimental colitis, such as T-cell- and dextran sulfate sodium (DSS)-induced colitis.^17,18^ However, some human clinical studies have reported mixed or no clear beneficial effects of SCFA-based therapies, suggesting varied levels of benefit depending on patient characteristics and treatment regimens.^19,20^ Ffar2 (GPR43) plays an important role in increasing barrier immunity to control invading microbes in gut tissues.^6^ SCFAs, however, can exacerbate acute colitis in animals induced with DSS or 2,4,6-trinitrobenzene sulfonic acid.^21,22^ Moreover, DF and SCFAs suppress chronic colitis and inflammation-associated colon cancer development.^23,24^ Beyond colitis, SCFAs are also implicated in ameliorating autoimmune neuroinflammation, kidney inflammation, and atherosclerosis.^25–27^ The protective effects of SCFAs are likely to be mediated through both tissue cells and immune cells, including epithelial cells, myeloid cells, T cells, B cells, and ILCs.

It should be noted that some of the anti-inflammatory effects of DF could be mediated by non-SCFA pathways. Certain functions of DF are mediated by physical properties, such as bulking fecal content and binding ions, biomolecules, and bacteria.^28,29^ Moreover, DF can contain other biologically active plant materials, which often have antioxidant and other protective activities. For example, ferulic acid, a phenolic compound in some plant cell walls, is released upon DF degradation by bacteria and can exert anti-inflammatory and other beneficial effects, in part, through its antioxidant properties.^30,31^ The effect of DF on microbiome composition is also significant in suppressing chronic inflammation and metabolic diseases.^32–34^

## Production and distribution of SCFAs in the body

SCFAs are mainly produced by commensal microbes as the end products of carbohydrate fermentation under anaerobic conditions in the colon (Fig. 1). SCFAs are incompletely oxidized metabolites and water soluble due to their short hydroxyl carbon backbones that contain fewer than six carbons. SCFAs are distinguished from hydrophobic medium-chain (C6-12) and long-chain (>C13) fatty acids. While DF (commonly called prebiotics) is the major source of SCFAs,^35^ other nutrients, such as proteins and peptides, can be metabolized to produce SCFAs, albeit at low levels.^36^ In this regard, proteins are the major source of minor volatile SCFAs, such as isobutyrate and isovalerate.^37^ While not a major source, C2 can also be produced from alcohol in host cells.^38^ Digestion-resistant oligofructose, inulin, pectin, and arabinoxylan are good prebiotics that are fermented by microbes to produce C2, C3, and C4.^39,40^ Cellulose, lignin, and chitin are types of insoluble dietary fiber; therefore, their bioavailability for the production of SCFAs is relatively low compared to that of soluble dietary fiber in the gut. Moreover, host carbohydrate biopolymers, such as mucins, can be fermented by certain microbes in the context of DF deficiency, leading to loss of the protective mucous layer.^41^

In the proximal colon of humans, the luminal concentrations of C2, C3, and C4 reach as high as ~130 mmol/kg of luminal content.^42^ The SCFA concentration in the distal colon is lower but still high at ~80 mmol/kg, and the concentration in the small intestine is ~15 mmol/kg. The lower part of the small intestine, particularly the last segment (the ileum), has significant levels of SCFAs. A significant portion of the SCFAs produced in the colon are absorbed into colonocytes.^43,44^ Much of the absorbed SCFAs are used by colonocytes, but some reach the blood by passive diffusion and active transport through solute transporters. The portal vein that moves absorbed nutrients from the intestine to the liver maintains fairly high SCFA concentrations at ~250 μM for C2, 20–200 μM for C3, and 15–65 μM for C4.^45^ SCFAs are also detectable in the peripheral blood at 20–150 μM for C2, 1–13 μM for C3, and 1–12 μM for C4 depending on the host condition. Comparable levels of SCFAs are present in mouse blood.^45^ These blood SCFA concentrations are considered high enough to affect host cells throughout the body.

Microbes are highly heterogeneous in their SCFA-producing capacity.^46,47^ The optimal diversity of commensal microbes, promoted by high levels of DF consumption and good health, leads to enrichment of SCFA producers.^31^ These microbes have polysaccharide utilization loci (PULs), which encode enzymes that recognize and degrade complex carbohydrates. PUL gene products allow microbes to make mono- and disaccharides from DF and other carbohydrates. These saccharides are utilized by microbes to produce SCFAs. Microbes with PULs may or may not produce SCFAs themselves because additional enzymes are required to ferment sugars into SCFAs. Most enteric and acetogenic bacteria produce C2 via the reductive acetogenesis process.^48^ Bacteria metabolize sugars to produce C3 through several different pathways, including the succinate, acrylate, and propanediol pathways.^49^ The succinate pathway is the preferred pathway for Bacteroidetes and some Firmicutes species. C4 is produced from acetoacetyl-CoA, which is produced from two molecules of acetyl-CoA. Butyryl-CoA:acetate CoA-transferase generates C4 from butyryl-CoA. *Roseburia, Eubacterium*, *Anaerostipes*, and *Faecalibacterium prausnitzii* species have butyryl-CoA:acetate CoA-transferase, which extends acetyl-CoA to produce C4.^36,49^ Another pathway to produce C4 is via phosphotransbutyrylase and butyrate kinase. For example, certain Coprococcus species and many Clostridium species in the Firmicutes family have butyrate kinase to produce C4.^50^

SCFA production can be altered by changes in the host condition, such as alterations in diet and the health status. Diets rich in dietary fiber, of course, increase SCFA production in the gut and increase SCFA levels in the blood. It has been reported that infection by helminths increases SCFA production by enriching *Trichinella spiralis*, a SCFA-producing species.^51^ This could benefit parasites because SCFAs suppress Th2 or antihelminth immune responses. In contrast, it has been reported that infection by influenza virus decreases intestinal SCFA production, leading to increased superinfection by pneumococci in the lungs.^5^ Thus, infection can alter SCFA production. The potentially distinct effects of SCFAs on the immune responses to different pathogens will be discussed later.

## Cellular uptake and intracellular functions of SCFAs

**Fig. 2:**
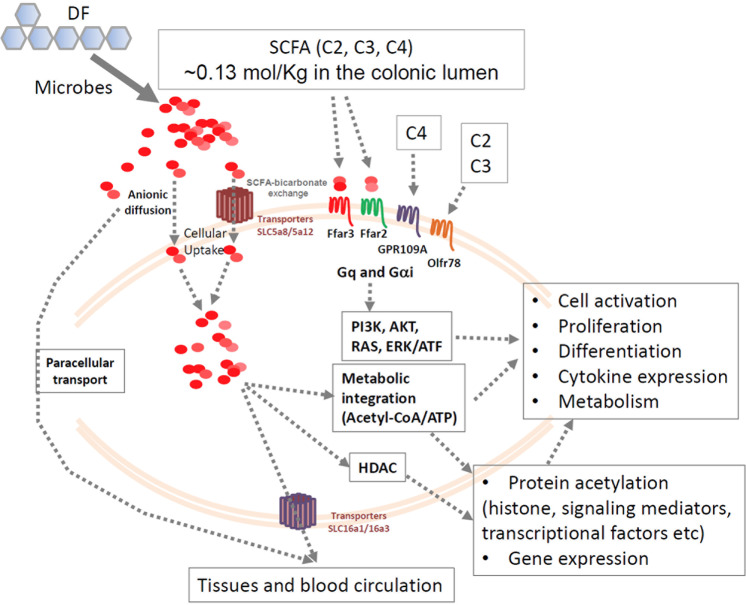
Transport, G-protein-coupled receptors, and intracellular functions of SCFAs. SCFAs are imported into colonocytes and tissues via several transporters and paracellular transport. Intracellular SCFAs are utilized within colonocytes, but some are further transported to the blood circulation. SCFAs activate cell-surface GPRs, such as Ffar2, Ffar3, GPR109A, and Olfr78, to activate signaling pathways, such as the PI3K, AKT, RAS, and ERK1/2 pathways. These signals crosstalk and boost cytokine signaling to induce cell activation and proliferation. SCFAs, particularly C3 and C4, are effective HDAC inhibitors and induce protein acetylation, which regulates cell activation and gene expression. SCFAs are also readily converted into acetyl-CoA to fuel the TCA cycle for the production of ATP and metabolic building blocks and to further support protein acetylation activity

SCFAs enter cells through passive diffusion and carrier-mediated absorption through solute transporters (Fig. 2). The major transporters include SLC5a8 (also called sodium-coupled monocarboxylate transporter 1) and SLC16a1 (monocarboxylate transporter 1).^52–55^ These transporters can transport SCFAs and related metabolites, such as ketone bodies, lactate, and pyruvate, into cells.^52,53,56–66^ Other transporters include SLC16a3 and SLC5a12.^67^ SLC5a8 and SLC5a12 are expressed in the apical membrane, whereas SLC16A3 is expressed in the basolateral membrane. SLC16a1 is expressed in both the apical and basolateral membranes.^67^ These transporters allow efficient transport of SCFAs from the gut lumen into colonocytes and lamina propria and eventually to the blood.

SCFAs are important nutrients for host cells. A significant portion of estimated calories come from SCFAs produced in the colon.^68^ It has been estimated that ~70% of the energy required to support colonocytes comes from SCFAs.^69^ SCFA metabolism in the intestine, liver, and muscles facilitates the production of cholesterol, long-chain fatty acids, glucose, glutamine, and glutamate.^70^ It is expected that SCFAs are metabolized by many cell types, including immune cells, which has the potential to support cell activation and functional maturation. For example, B cells can take up SCFAs to increase acetyl-CoA levels for fatty acid synthesis and fuel the TCA cycle (tricarboxylic acid cycle).^45^ This function of SCFAs is important for immune cell differentiation, as described in detail in the next sections.

SCFAs have long been known as natural inhibitors of HDACs.^71^ C4 and C3 have higher HDAC-inhibiting activity than C2.^72–74^ SCFAs directly inhibit class I/II HDACs.^73,75,76^ The activity of class III HDACs, such as Sirt1, may be indirectly downregulated by SCFAs through gene expression regulation.^77^ Histone acetyl transferases (HATs) induce acetylation of proteins, whereas HDACs remove acetyl groups, thus antagonizing the acetylation activity of HATs.^78^ With effects on both acetyl-CoA production and HDAC inhibition, SCFAs effectively promote protein acetylation, which affects the functions of many proteins, including histones and their activity related to chromosomal conformation and gene expression.^79–81^ SCFA-mediated HDAC inhibition promotes cell type-specific biological processes, and this function is largely independent of cell-surface SCFA receptors.

## Cell-surface GPRs sense SCFAs in the extracellular space

SCFAs in extracellular tissue spaces are sensed by several GPR (Fig. 2). Ffar2 and Ffar3 (GPR41) sense the presence of C2, C3, and C4 with somewhat different sensitivities.^82,83^ The agonistic activity of C2 and C3 specific for Ffar2 starts at ~10 μM and peaks at 10 mM for Ffar2-overexpressing Chinese hamster ovary cells.^84^ C4 and other longer chain SCFAs can activate Ffar2 only at high (millimolar) concentrations. Thus, Ffar2 is activated more readily by C3 and C2 than C4.^84^ In contrast, Ffar3 is activated better by C3 and C4 than C2. Ffar3 is expressed in apical enterocytes and basolateral enteroendocrine cells in the human colon.^85^ Enteroneuronal cells and sympathetic ganglia express Ffar3, which is relevant for the regulatory effect of SCFAs on gut motility.^86,87^ Ffar3 is also expressed by cells in adipose and pancreatic tissues and by renal smooth muscle cells.^83,88–90^ This expression pattern of Ffar3 in various cell types is in line with the effect of SCFAs on the production of gut hormones, such as glucagon-like peptide 1, peptide YY, cholecystokinin, and leptin, to regulate metabolism and obesity. In the immune system, Ffar3 is expressed by thymic medullary epithelial cells, B cells (follicular, GC, and B1b), spleen CD8^+^ dendritic cells (CD8^+^ DCs), neutrophils, Nkp46^+^ ILC3s, and blood monocytes (Table 1). Ffar2 is also expressed on enterocytes, mucosal mast cells, and enteroendocrine cells.^91,92^ Ffar2 is also expressed by leukocytes, such as eosinophils, basophils, neutrophils, monocytes, and DCs.^93^ RNA-seq data indicate that thymic medullary epithelial cells, B cells, and Nkp46^+^ ILC3s also express Ffar2 (Table 1). GPR109A senses C4 and niacin (vitamin B3).^94,95^ GPR109A expression has been detected in neutrophils and macrophages (Table 1). Another SCFA receptor, Olfr7, senses C2 and C3. It is expressed in the kidneys by renal afferent arterioles and autonomic nervous cells.^96–98^ This is consistent with the effects of C2 and C3 on renin production and the regulation of blood pressure. In the immune system, Olfr78 mRNA is expressed by T cells (CD8, γδ, and NKT), B cells (follicular and GC), and ILC2s (Table 1). SCFA-sensing GPRs play comprehensive roles from metabolic to neuronal and immune regulation through their fast-acting signaling. More studies are required to determine the cell-specific functions of these receptors. It is expected that these receptors are probably activated in intestinal tissues due to the high SCFA levels, but we need to better understand when and where these receptors are activated in systemic tissues where SCFA levels are relatively low.

**Table 1:** Expression of SCFA receptors or transporters in the immune system^a^

Molecules	Cell types that express the molecules at the mRNA level with relative expression levels indicated
Ffar2/GPR43	ILC3s > Neutrophils > MZ B cells > Pre-T cells (double-negative thymocytes) > DCs (spleen) > ILC2s > Alveolar macrophages
Ffar3/GPR41	Thymic medullary epithelial cells > Follicular B cells > GC B cells > DCs (CD8) > B1b cells ≈ Neutrophils ≈ Nkp46^+^ ILC3s ≈ Blood monocytes
Gpr109a/Hcar2	Neutrophils > Red pulp macrophages > Alveolar macrophages
Olfr78	CD8 T cells (memory) > γδ T cells > Follicular B cells > GC B cells > NKT cells > ILC2s
Slc5a8	Thymic medullary epithelial cells >> NKT cells = Neutrophils
Slc5a12	Thymic medullary epithelial cells > Mast cells > NK cells ≈ NKT cells = ILC3s > ILC2s ≈ Thymocytes (double positive) ≈ pDCs > Macrophages > DCs (CD8)
Slc16a1	Pro-B cells > Mast cells ≈ Thymocytes (double-negative and positive) > Naive CD8 T cells > Effector CD8 T cells ≈ GC B cells ≈ NKT cells ≈ ILC2s

^a^Retrieved from the RNA-seq data deposited in the Immunological Genome Project^175–183^

## Distinct regulation of ILC subsets by SCFAs and GPR signaling

ILCs are present throughout the body, including barrier tissues, and appear to be a key target of regulation by microbial metabolites.^99^ ILCs do not express antigen receptors but are similar to T cells in the expression of cytokines and master transcription factors (i.e., RORγt for ILC3s, Gata3 for ILC2s, and T-bet for ILC1s). They are primarily activated by cytokines produced by various tissue and myeloid cells in an ILC group-specific manner.^100^ ILCs originate from progenitors in the fetal liver and adult bone marrow (BM).^101–103^ The development of common ILC progenitors in the BM requires IL-7 and a number of transcription factors, such as Id2, TOX, and Nfil3.^104–108^ ILCs include group 1 (NK cells and non-NK ILC1s), group 2 (ILC2s), and group 3 (ILC3s).^109^ ILC1s produce IFNγ. ILC2s produce IL-5, IL-9, and IL-13. ILC3s produce IL-22, IL-17A/F, and GM-CSF. ILC3s are subdivided into lymphoid tissue-inducer (LTi) cells and other ILC3s, which are further divided into natural cytotoxicity receptor (NCR)^+^ and NCR^−^ ILC3 subsets.^51,52^ ILC1s respond to and control infection by obligate intracellular pathogens (i.e., viruses, Salmonella, and *Toxoplasma gondii*). ILC2s respond to helminth infection. ILC3s respond to extracellular pathogens (bacteria and fungi) and are effective in clearing pathogens. In addition, ILCs, such as ILC2s and ILC3s, stimulate tissue remodeling and repair^110,111^ and regulate adaptive immunity.^112,113^ Moreover, ILC2s induce beiging of white adipose tissue for lipolysis.^114,115^ ILC3s are important regulators of intestinal barrier immunity and regulate the microbiota.^109,116–118^

In general, peripheral ILC activity is profoundly affected by the microbiota. In particular, ILC3 activity is highly regulated by the microbiota.^119–121^ There are several mechanisms by which microbes regulate ILCs. First, the microbiota stimulates epithelial cells and antigen-presenting cells, such as macrophages and DCs.^122^ Triggering TLRs on these cells can induce ILC-stimulating cytokines.^123^ The microbiota increases the numbers and activity of ILC3s by inducing the expression of IL-1β and IL-23.^123–126^ On the other hand, Thymic Stromal Lymphopoietin/TSLP, IL-33, and IL-25 trigger ILC2 proliferation and functional activation, whereas type I/II IFNs suppress ILC2 responses.^127,128^ Commensal microbes induce the expression of IL-12, IL-15, and IL-18, which increases ILC1 activity.^116,121,129^ While there is no clear evidence that microbes directly regulate NK cells and ILC1s, the activity of these cells may be indirectly affected by microbiota-stimulated mononuclear phagocytes, which produce activating cytokines, such as type I interferons.^120^

It has been observed that the metabolites produced by commensal bacteria greatly influence ILCs. Microbial metabolites are diverse, including those derived from carbohydrates, proteins, phytochemicals, and host biomolecules.^1^ Some of these metabolites activate aryl hydrocarbon receptor (AhR), pregnane X receptor, farnesoid X receptor, and TGR5, which are differentially expressed by various host cells. For example, indole-3-acetate (I3A) agonizes AhR, which is a transcription factor that induces certain groups of genes, including those that encode enzymes that metabolize toxic chemicals or factors that regulate cell differentiation and activation. I3A increases NCR^+^ and LTi ILC3 responses in an AhR-dependent manner.^124,126,130^

**Fig. 3:**
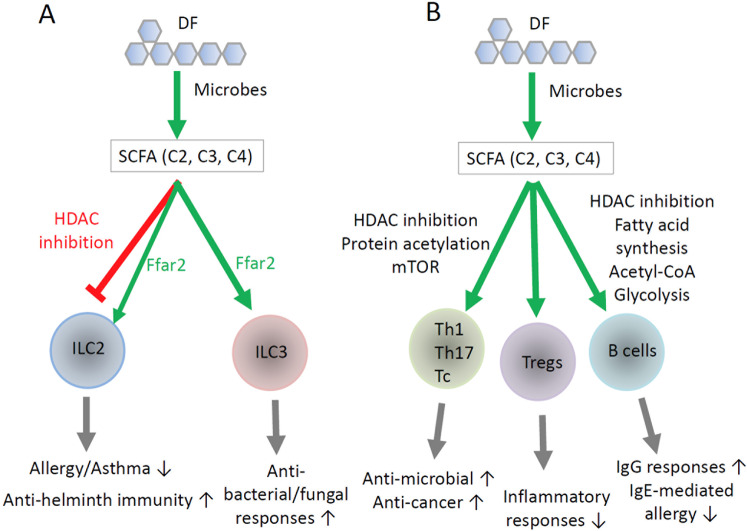
Direct regulation of ILCs, T cells, and B cells by SCFAs. **A** SCFAs differentially regulate ILC2s and ILC3s. In general, SCFAs increase ILC3 activity, while they suppress ILC2 activity. Ffar2 signaling in ILC2s and ILC3s triggers PI3K, AKT, and mTOR activity to promote cell proliferation and activation. The effects of SCFAs and GPR43 on ILCs are not identical. While Ffar2 signaling increases the activity of both ILC2s and ILC3s, SCFAs increase ILC3 function but suppress ILC2s. This implies that the GPR signaling vs. intracellular functions of SCFAs can play distinct roles in regulating ILC2s. **B** DF and SCFAs support the activity of T helper cells, T cytotoxic cells, Tregs, and B cells. A key function of SCFAs is increasing protein acetylation and cellular metabolism in T and B cells. This influences naive T-cell differentiation into Th1 cells, Th17 cells, and Tregs. SCFAs do not polarize naive T cells undergoing differentiation themselves but costimulate T cells along with cytokines and TCR activation to vigorously generate effector Th1 and Th17 cells. In a TGFβ-rich environment in a steady state, such as in the intestine, SCFAs induce the generation of IL-10-producing Tregs. SCFAs also boost the effector function of CD8 T cells to promote cytotoxic activity. SCFAs also energize B cell activation and differentiation into GC B cells and plasma cells for the production of IgG and IgA but suppress the production of IgE. The regulatory effects of SCFAs on immune cells reach beyond the intestine. While Ffar2 is important in the regulation of ILCs, the intracellular effects of SCFAs, such as those mediated by acetyl-CoA and HDAC inhibition, play major roles in the regulation of T and B cells. Overall, SCFAs support the effector functions of lymphocytes to defend against microbial pathogens and cancer but can weaken immunity against helminths. SCFAs can also exert anti-inflammatory and antiallergy functions, in part, by strengthening gut barrier immunity, supporting Treg activity, and suppressing IgE production, mast cells, and ILC2s

As major carbohydrate metabolites, SCFAs can regulate ILCs.^13,131,132^ SCFA positively regulate intestinal ILC3s. Infection by extracellular bacteria, such as *Citrobacter rodentium* and *Clostridium difficile*, induces ILC3 responses, which are effective in clearing the infection (Fig. 3). Defective ILC3 responses have been observed in Ffar2-deficient mice infected by these pathogens.^132,133^ Colonic ILC3s express Ffar2, and Ffar2 agonism promotes ILC3 activity in the intestine.^132^ Chun et al. reported that Ffar2 increased AKT and STAT3 signaling and the numbers of IL-22^+^CCR6^+^ ILC3s. Another group reported that C2 is beneficial in ameliorating *C. difficile* infection.^133^ This amelioration is mediated by Ffar2-dependent recruitment of neutrophils and ILC3s. In this context, neutrophils highly express GPR43 and are chemotactically attracted to SCFAs. C2 also facilitates inflammasome activation to facilitate the release of IL-1β from neutrophils. Ffar2 signaling augments the expression of IL-1β receptor on ILC3s, resulting in Ffar2- and IL-1β-enhanced ILC3 responses. We also observed that Ffar2 supports the proliferation and function of colonic ILC3s.^134^ Ffar2 signaling also has a positive effect on ILC1s, but compared to that on ILC3s, the effect seems smaller. Ffar2 signaling costimulates cytokine signaling for robust ILC proliferation and activation. Key pathways that are boosted by Ffar2 signaling are the STAT3, STAT5, STAT6, and PI3K pathways. In this regard, mTOR activation, cell proliferation, and IL-22 production are enhanced by Ffar2 activation in ILC3s. A recent report indicated that SCFAs can also function as AhR agonists in intestinal epithelial cells.^135^ Thus, there is a possibility that the ILC3-boosting activity of SCFAs is mediated, in part, by their AhR activation function.

While Ffar2 signaling supports ILC2 activity, SCFAs contradictorily suppress overall ILC2 activity.^134^ Thus, the effects of SCFAs and Ffar2 signaling on ILC2s can be different, which may be because SCFAs regulate cells via several different mechanisms beyond cell-surface GPRs. Other groups have also reported that SCFAs suppress ILC2 responses and associated allergic responses in the lungs.^12,13^ The detailed mechanism remains speculative, but Ffar2-independent intracellular mechanisms are likely to be involved. In this regard, ILC2s express SCFA transporters to take up SCFAs for intracellular functions.^134^ SCFAs acetylate histones in ILC2s, and HDAC inhibition appears to be involved in this process.^12,13^ Administration of butyrate producers, such as *Clostridia butyricum* and *Clostridia sporogenes*, induce elevated levels of C3 and C4 in the lungs and decrease the numbers of IL-5/IL-13-producing ILC2s.^13^ Additionally, C4 administration suppresses the *Alternaria alternata* (an allergenic fungus) extract-induced ILC response and reduces lung allergy severity (Table 2).^12^ Thus, SCFAs regulate ILC subsets in a shared yet distinct manner through multiple mechanisms that involve GPR signaling and GPR-independent intracellular functions.

**Table 2:** Regulation of infection, inflammation, and allergy by DF and SCFAs

Diseases	Exacerbation	Protection	References
Infection by extracellular pathogens		*C. rodentium* *C. difficile*	^6,45,133^
Infection by intracellular pathogens		Influenza virus Respiratory syncytial virus *Listeria monocytogenes* *Salmonella typhimurium*	^3–5,184,185^
Infection by helminths	*Trichuris muris*		^7^
Colitis	Acute 2,4,6-trinitrobenzene sulfonic acid (TNBS)- or dextran sulfate sodium (DSS)-induced colitis	Chronic DSS- or azoxymethane (AOM)/DSS-induced colitis	^20–23,186,187^
Autoimmune diseases		Experimental autoimmune encephalitis Cuprizone-induced demyelination Chronic kidney disease Type I diabetes	^25,27,34,174,188,189^
Allergic diseases	Allergen-induced lung allergy/asthma, peanut-induced food allergy in mice		^10,12,13^
Other inflammatory diseases	Spontaneous SCFA-induced urethritis in mice	Atherosclerosis	^145,160^

## SCFAs support both the effector and regulatory functions of T cells depending on the host condition

Early work identified the potential of SCFAs in regulating cytokine production by T cells and other cells.^136–138^ Later, it was determined that SCFAs promote the activity of regulatory T cells (Tregs).^139^ Tregs are heterogeneous, including both FoxP3^+^ T cells and FoxP3^−^ T cells, which may or may not produce IL-10. FoxP3^+^ Tregs express CTLA4, TGF-β1, IL-35, galectin-1, granzymes, and other effector molecules to suppress major types of immune cells and thus prevent inflammatory diseases.^140^ While the exact phenotype of the increased Treg population established by SCFAs is equivocal, the consensus in the field is that SCFAs increase the activity of FoxP3^+^ T cells and IL-10 production.^141,142^ SCFAs, when administered orally, efficiently increase the numbers of Tregs in the colon. DF feeding can also increase Treg numbers in the intestines and lungs.^143^ In support of this role of SCFAs, SCFA-producing bacteria, such as certain Clostridia strains, support the generation of FoxP3^+^ T cells.^144^ Thus, SCFAs maintain tolerogenic T cells in the steady state to prevent potential inflammatory responses in the intestine (Fig. 3). The HDAC-inhibiting function of SCFAs increases histone acetylation to regulate gene expression. The *FoxP3* and *IL-10* gene loci are targets of such regulation in the steady state.^141,142^ A question that arises is how SCFAs selectively induce the expression of a few genes, such as *FoxP3* and *IL-10*. Indeed, this is not highly likely because SCFAs regulate a myriad of other genes in T cells undergoing activation.^72^ Moreover, indirect functions of SCFAs mediated through other cell types, such as DCs, are also important in inducing Tregs and IL-10 expression.

SCFAs also boost the generation of Th1 and Th17 cells during active immune responses.^72,145^ SCFAs promote Th1 and Th17 polarization in vitro in the presence of appropriate cytokines. Moreover, SCFAs support Th1 and Th17 responses not only in the intestine but also in systemic tissues, such as the spleen and lymph nodes, during *C. rodentium* infection (Table 2).^6^ It has also been reported that the adjuvant effect of cholera toxin involves the SCFA-Ffar2 axis.^146^ It appears that SCFAs boost T-cell responses in a manner dependent on host conditions or the immunological milieu (Fig. 3). In the steady state, SCFAs favor IL-10-mediated immune tolerance. However, during active immune responses, SCFAs help generate the effector T cells required to clear pathogens. This can also be applied to cytotoxic (CD8) T cells. SCFAs increase the cytotoxic activity and IL-17 production capacity of CD8 T cells.^147^ Moreover, C4 enhances the memory T-cell response upon antigen re-encounter.^148^ A major mechanism appears to involve HDAC inhibition and metabolic regulation rather than cell-surface GPRs. Indeed, most mature T cells hardly express SCFA-sensing GPRs, except Olfr78, which is expressed by some memory CD8 T cells (Table 1). A SCFA-related metabolite, β-hydroxybutyrate, is generated through ketogenesis by host cells and can epigenetically modify Lys 9 of histone H3 (H3K9) associated with certain genes, such as Foxo1 and Ppargc1a (which encodes PGC-1α). These factors upregulate the expression of the cytosolic phosphoenolpyruvate carboxykinase Pck1,^149^ which is required for optimal memory CD8 T-cell generation. It is important to note that SCFAs and related HDAC inhibitors are likely to affect many genes in CD8 T cells, the effect of which has yet to be studied. While the increased effector T-cell population can fight infections, there is a possibility that SCFAs may also increase potentially harmful inflammatory responses (Table 2). Indeed, chronic SCFA feeding induces Th17-mediated urethritis, resulting in hydronephrosis.^145^ Whether SCFAs exacerbate other chronic inflammatory diseases should be studied.

SCFAs profoundly affect the mTOR pathway in T cells and regulate cellular metabolism.^72^ SCFAs are readily converted to acetyl-CoA, which is a key metabolic currency that fuels major metabolic processes, such as the mitochondrial TCA cycle, fatty acid synthesis, and protein acetylation. The TCA cycle produces ATP and metabolic building blocks. Increased levels of ATP fuel many cellular activities and relieve AMP-induced suppression of mTOR activation.^150^ Appropriate regulation of mTOR activity is critical for normal T-cell differentiation into effector vs. regulatory T cells. For example, polarization of Th1 and Th17 cells requires high mTOR activity.^151,152^ In T cells, SCFAs induce acetylation of S6K, which is downstream of the mTOR pathway.^72,153^ This is likely induced by the HDAC-inhibiting activity of SCFAs. Thus, S6K could be a potential molecular target of SCFAs involved in increasing mTOR activity in T cells. In general, mTOR activity promotes effector T cells at high levels but promotes FoxP3^+^ Treg generation at low levels. Increased mTOR activity and ATP levels induced by SCFAs support the generation of Th1 cells, Th17 cells, and IL-10^+^ T cells.

SCFAs regulate certain tissue cells and antigen-presenting cells through HDAC and GPR triggering. DCs and macrophages are regulated by SCFAs, indirectly regulating T-cell activity. SCFAs suppress not only the hematopoiesis of myeloid DCs but also the functional maturation of DCs.^21,154–156^ SCFAs also suppress the upregulation of the expression of MHC II, CD80, CD86, and IL-12, which are important for activating T cells and generating Th1 cells.^157^ It has been reported that SCFA-treated DCs have decreased IL-12 production but increased IL-23p19 production.^21^ Signaling through GPR109a and Ffar2 induces Treg-inducing DCs.^158^ SCFAs also generate tolerogenic macrophages that induce IL-10 production in T cells in a GPR109a-dependent manner.^158^ It has also been reported that C4 conditions macrophages to decrease the expression of inflammatory cytokines, such as TNF-α, MCP-1, and IL-6, and this effect is likely to be mediated via HDAC inhibition.^159,160^ Thus, the direct and indirect effects of SCFAs can coordinately support both effector T cells and regulatory T cells in various host conditions.

## SCFAs regulate antibody production

Commensal microbiota species regulate B cell responses and antibody production. In germ-free mice and mice treated with antibiotics, the production of IgG and IgA in response to pathogens is decreased.^45,161,162^ Moreover, DF feeding generally has positive effects on blood and gut luminal levels of antibodies, such as IgG and IgA.^163–165^ Similarly, shorter prebiotics, such as galacto-oligosaccharides, increase IgA production.^10^ A mechanism of B cell regulation by DF appears to be mediated by enrichment of certain microbes that promote B cell responses. DF enriches DF-utilizing microbes, leading to increased SCFA production. In this regard, certain probiotics, such as *Lactobacillus* and *Bifidobacteria* species, increase the production of IgG and IgA. SCFAs, when administered in the drinking water, increase antibody production.^45^ SCFAs boost B cell differentiation into germinal center (GC) B cells and plasma cells in secondary lymphoid tissues, such as Peyer’s patches, the mesenteric lymph nodes and the spleen. SCFAs enhance the effect of the activation signals from B cell receptor and cytokine receptors when triggered by IL-5, IL-6, and IL-10.^45^ While DF and SCFAs increase the production of IgG and IgA, they suppress IgE production. This is in line with the decrease in IgE-mediated allergic responses mediated by DF and SCFAs.^10^ However, DF and SCFAs exacerbate helminth infections and related inflammatory responses (Table 2).^7^ This is because IgE is a key effector molecule in defense against helminth infection, and therefore, decreased IgE levels can weaken antihelminth immune responses.

B cells undergo activation, proliferation, differentiation, and antibody secretion, and these processes require efficient production of energy and cell constituents, such as lipids and proteins.^166^ B cells require glycolysis, oxidative phosphorylation, and fatty acid synthesis during activation. Glycolysis is particularly important for the survival of GC B cells, and oxidative phosphorylation is relatively more important for the maintenance of plasma cells.^167,168^ B cells require fatty acid synthesis to become plasma cells.^169,170^ SCFAs increase the levels of Acetyl-CoA and ATP. Acetyl-CoA fuels the TCA cycle to generate ATP in B cells. This leads to activation of mTOR, which is required for normal B cell activation to differentiate into plasma cells.^45^ SCFAs also participate in fatty acid synthesis in B cells through acetyl-CoA, which is converted to malonyl-CoA to enter the fatty acid synthesis process. Thus, SCFAs have the capacity to support B cell metabolism upon B cell activation. Along with the metabolic effect, HDAC inhibition by SCFAs is thought to play a key role because trichostatin A, which is a pan-HDAC inhibitor, can mimic the B cell-boosting activity of SCFAs. SCFAs induce epigenetic regulation of key genes involved in metabolic regulation and cell differentiation, such as *Aicda, Xbp1*, and *Irf4*, all of which play key roles in B cell differentiation and antibody production.^45^ SCFAs increase the number of Tfh cells, which are specialized T helper cells that support B cell differentiation into plasma cells.^45^ SCFAs also increase the levels of epithelial cytokines, such as IL-6,^6^ which stimulates B cells for antibody production.^171^

B cells express GPRs to sense SCFAs (Table 1), and it has also been reported that mice deficient in Ffar2 have a relatively low intestinal IgA response.^172^ Thus, DF and SCFAs can regulate B cells via a number of direct and indirect mechanisms. However, SCFAs can suppress activation-induced deaminase expression and IgG1 production at high SCFA concentrations, and mice fed a diet low in insoluble/soluble DF have increased IgG1 production over those fed high levels of DF.^173^ Overall, DF and SCFAs are significant factors in regulating host antibody responses (Fig. 3).

## Concluding remarks

The broad and lymphocyte-specific regulatory functions of SCFAs have significant impacts on the immune system. ILC3s, T cells, and B cells in the intestine are the primary targets of regulation by SCFAs because the levels of SCFAs are highest in the gut, where SCFAs support the activity of these lymphocytes to promote balanced intestinal immunity and immune tolerance. These effects of SCFAs on lymphocytes appear to work together with those on epithelial cells and myeloid cells to strengthen intestinal barrier immunity, regulate microbes, and prevent harmful inflammatory responses. A significant portion of gut-derived SCFAs are transported out of the gut to impact other organs; therefore, SCFAs affect immune cells beyond the cells in the gut. Indeed, it has been reported that DF and SCFAs increase immune responses in the lungs during viral infection and regulate inflammatory responses in the central nervous system. Moreover, SCFAs regulate systemic lymphocyte responses mediated by CD4 T cells, CD8 T cells, B cells, and ILCs during infection.

Because many children and adults, particularly those in certain demographic groups in developed countries, do not consume enough dietary fiber, SCFA deficiency has become a potential health problem. SCFA deficiency can result in weak or imbalanced immunity and increase infection by bacterial and viral pathogens or perhaps enhance susceptibility to autoimmune diseases. For example, decreased blood levels of SCFAs were observed in patients with chronic inflammatory diseases such as long-term multiple sclerosis.^174^

While SCFAs are beneficial, SCFAs have the potential to exacerbate certain infections and inflammatory diseases. For example, infection by helminths is likely to be worsened by DF deficiency because SCFAs decrease IgE production and ILC2 activity and suppress mast cells. On the other hand, SCFAs exert beneficial effects on allergic responses because they can decrease the activity of the same immune effectors. It has been documented that chronic elevation of SCFA levels higher than physiological levels can cause T-cell-induced inflammatory responses in the renal system.^145^ To make the topic even more complex, the functions of SCFAs and their GPRs are not always equivalent because SCFAs can regulate cellular responses in SCFA receptor-independent manners, and many cell types lack or hardly express SCFA-sensing GPRs. This suggests that the functional SCFA system, which is composed of DF, microbes, SCFAs, transporters, receptors, HDACs, cellular metabolism, and downstream signaling pathways, can regulate the immune system in many different ways. Further studies are required to dissect these regulatory mechanisms and determine their impacts on the immune system in various host conditions.
